# Primary and Orthotopic Murine Models of Nasopharyngeal Carcinoma Reveal Molecular Mechanisms Underlying its Malignant Progression

**DOI:** 10.1002/advs.202403161

**Published:** 2024-07-25

**Authors:** Xudong Wan, Yuantao Liu, Yiman Peng, Jian Wang, Shu‐mei Yan, Lu Zhang, Wanchun Wu, Lei Zhao, Xuelan Chen, Kexin Ren, Haicheng Long, Yiling Luo, Qin Yan, Lele Zhang, Dengzhi Lei, Pengpeng Liu, Shujun Li, Lihui Liu, Linjie Guo, Jiajia Du, Mengsha Zhang, Siqi Dai, Yi Yang, Hongyu Liu, Nianyong Chen, Jinxin Bei, Lin Feng, Yu Liu, Mu‐sheng Zeng, Chong Chen, Qian Zhong

**Affiliations:** ^1^ Division of Thoracic Tumor Multimodality Treatment State Key Laboratory of Biotherapy and Cancer Center West China Hospital Sichuan University Chengdu Sichuan 610041 China; ^2^ State Key Laboratory of Oncology in South China Collaborative Innovation Center for Cancer Medicine Department of Experimental Research Sun Yat‐sen University Cancer Center Sun Yat‐sen University Guangzhou Guangdong 510060 China; ^3^ Department of Biotherapy State Key Laboratory of Biotherapy and Cancer Center West China Hospital Sichuan University Chengdu Sichuan 610041 China; ^4^ Department of Hematology West China Hospital Sichuan University Chengdu Sichuan 610041 China; ^5^ Department of Gastroenterology West China Hospital Sichuan University Chengdu Sichuan 610041 China; ^6^ Department of Otolaryngology West China Hospital Sichuan University Chengdu Sichuan 610041 China; ^7^ Frontiers Medical Center Tianfu Jincheng Laboratory Chengdu Sichuan 610212 China

**Keywords:** Epstein‐Barr virus, metastasis, primary and orthotopic murine NPC models, TGFBR2

## Abstract

Nasopharyngeal carcinoma (NPC), a squamous cell carcinoma originating in the nasopharynx, is a leading malignancy in south China and other south and east Asia areas. It is frequently associated with Epstein‐Barr virus (EBV) infection, while there are also some NPC patients without EBV infection. Here, it is shown that the EBV+ (EBV positive) and EBV‐ (EBV negative) NPCs contain both shared and distinct genetic abnormalities, among the latter are increased mutations in *TP53*. To investigate the functional roles of NPC‐associated genetic alterations, primary, orthotopic, and genetically defined NPC models were developed in mice, a key tool missed in the field. These models, initiated with gene‐edited organoids of normal nasopharyngeal epithelium, faithfully recapitulated the pathological features of human disease. With these models, it is found that *Trp53* and *Cdkn2a* deficiency are crucial for NPC initiation and progression. And latent membrane protein1 (LMP1), an EBV‐coding oncoprotein, significantly promoted the distal metastasis. Further, loss of *TGFBR2*, which is frequently disrupted both in EBV‐ and EBV+ NPCs, dramatically accelerated the progression and lung metastasis of NPC probably by altering tumor microenvironment. Taken together, this work establishes a platform to dissect the genetic mechanisms underlying NPC pathogenesis and might be of value for future translational studies.

## Graphical abstract

A comparative genomics analysis of EBV+ and EBV‐ NPCs through WES to reveal their both shared and distinct genetic abnormalities is reported and created a serial of primary, orthotopic, and genetic driver‐defined NPC mouse models initiated with gene‐edited normal nasopharyngeal organoids to explore molecular mechanisms underlying NPC tumorigenesis.

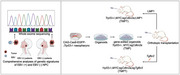

## 1 Introduction

Nasopharyngeal carcinoma is a unique and the most common head and neck malignant tumor in southeastern Asia, and it presumably originates from the nasopharyngeal epithelium and frequently associated with Epstein‐Barr virus infection.^[^ ^1^, ^2^ ^]^ While the early‐stage disease is sensitive to radiotherapy and gemcitabine‐cisplatin based chemotherapy, the advanced NPCs often display distal metastases and respond poorly to these treatments.^[^ ^3^, ^4^ ^]^ Developing new diagnostic and treatment strategies requires a better understanding of the molecular mechanisms underlying the process of NPC tumorigenesis.

There are accumulating evidence suggesting that genetic alterations, Epstein‐Barr virus infection and environmental factors, together with many others, might be involved in the tumorigenesis of NPC.^[^ ^5^, ^6^ ^]^ Frequent genetic alterations associated with NPCs include both chromosome abnormalities and gene mutations.^[^ ^7^, ^8^, ^9^ ^]^ And consistently, *CCND1* and *MYC* are frequently amplified while *TP53* is the most frequently mutated gene in NPCs, together with many others, such as *ARID1A, KMT2C* and *KMT2D*, whose functions in NPCs are less understood.^[^ ^7^, ^10^, ^11^, ^12^ ^]^ Chromosome copy number variations are also common, such as chromosome 3p and 9p deletions, which occur in 20%–75% and 20%–50%, respectively, of NPC patients.^[^ ^6^, ^7^ ^]^ And *CDKN2A/B* loss through chromosome 9p deletions is important for the proliferation of NPC cells.^[^ ^13^ ^]^ At the same time, more than 90% of NPCs are associated with EBV, which has been shown to be tumorigenic in NPC and also many other human malignancies including Hodgkin's B lymphoma and gastric carcinoma possibly due to expressing viral oncogenes latent membrane proteins (LMP) and EBV‐determined nuclear antigens (EBNA).^[^ ^5^, ^14^, ^15^ ^]^ Of note, there are a portion of NPCs which are EBV.^[^ ^16^ ^]^ And so far, the genetic signature of the EBV‐ NPCs was largely unexplored.

To understand the potential functions and underlying mechanisms of these genetic and non‐genetic alterations in the pathogenesis of this disease, NPC models that can faithfully recapitulate the human disease are essential. Currently, NPC cell lines are still the most used, which often suffer from genetic shift and the lack of proper systematic and microenvironmental factors.^[^ ^17^ ^]^ Recently, a few patient‐derived xenografts (PDX) have been generated with tumor tissues from NPC patients. It has been shown that PDX can maintain the genetic alterations and pathological features of NPC and thus is valuable for testing the responses of potential treatments.^[^ ^18^, ^19^ ^]^ Cancer organoids, a 3D culture of tumor cells that can represent the heterogeneity of cancer cells, are also cultured from NPC tissues.^[^ ^20^ ^]^ However, all these current NPC models started with fully transformed NPC cells and so far, there have been no primary NPC models reported. Therefore, current NPC models cannot reproduce the process of NPC tumorigenesis, which is critical for investigating the tumorigenesis‐driving functions and mechanisms of the disease‐associated factors in this malignancy.

In this study, we reported comparative genetic analyses of EBV+ and EBV‐ human NPC samples through whole‐exome sequencing (WES) to reveal the common and unique characteristics of EBV+ and EBV‐ NPCs. We generated primary and orthotopic NPC models starting from normal nasopharyngeal epithelium with NPC related genetic drivers, a capable strategy that we have successfully applied for lung cancer,^[^ ^21^ ^]^ gastric cancer,^[^ ^22^ ^]^ bladder cancer,^[^ ^23^ ^]^ and others.^[^ ^24^ ^]^ With these models, we further investigated the biological functions of multiple important genetic alterations, including *Trp53, Cdkn2a*, and *Tgfbr2* loss and LMP1 overexpression, during NPC initiation and development. Accordingly, our work establishes a novel platform to investigate the molecular mechanisms underlying NPC pathogenesis.

## 2 Results

### 2.1 Comprehensive Analyses of Genetic Signatures of EBV+ and EBV‐ NPC using Whole‐Exome Sequencing

**Figure 1:**
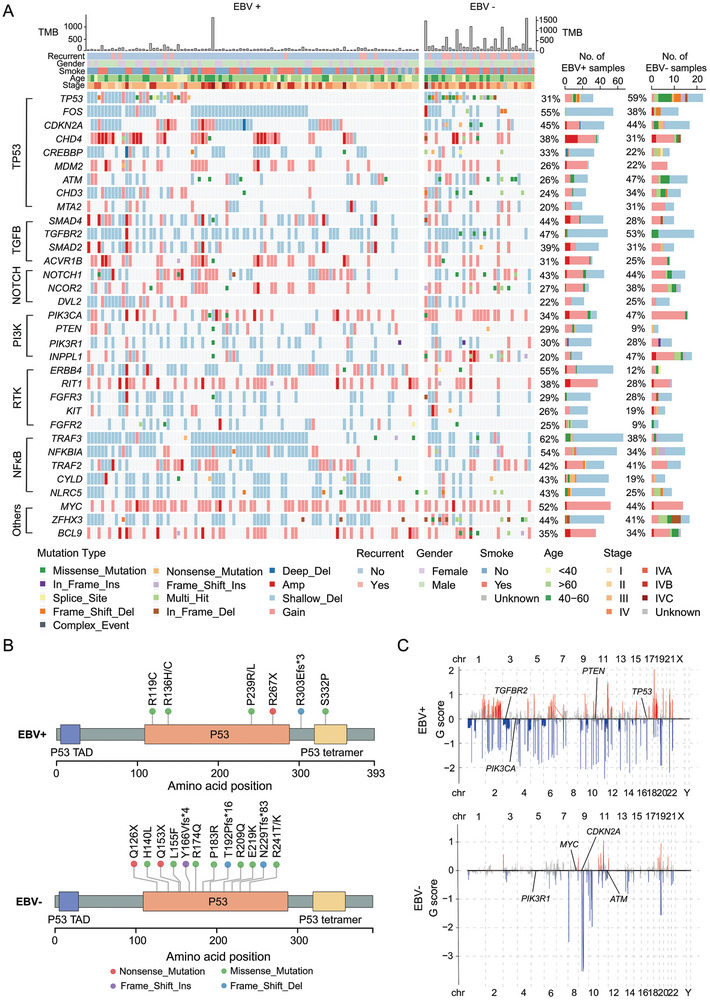
The genomic alteration landscape of both EBV+ and EBV‐ NPCs. A) Panoramic waterfall plot of SNVs and CNVs in EBV+ and EBV‐ NPC patients. Important pathways and mutations are presented, and for each case, the recurrence, smoking status, gender, patient age, and clinical stage are displayed in the corresponding upper left corner. B)The mutation status of the *TP53* gene in EBV+ and EBV‐ patients is illustrated through lollipop plot. C) Chromosome arm‐level CNV frequencies in EBV+ and EBV‐ NPC patients. CNV data processed by GISTIC2 and regions with FDR q < 0.1 are considered significant. (Red: amplifications, Blue: deletions).

We conducted comprehensive sequencing studies on samples obtained from 32 patients who were from high‐risk areas for NPCs but tested negative for EBV DNA copy number and ISH of EBERs (**Figure** **1**A; Figure S1A and Table S1, Supporting Information). Additionally, we included an additional 96 EBV+ NPC patients from previous studies for comparison.^[^ ^25^ ^]^ Clinical data analysis revealed that in these high‐risk areas, EBV‐ patients exhibited a higher recurrence rate compared to their EBV+ counterparts. Furthermore, EBV‐ patients displayed a wider age distribution, suggesting potential differences in disease characteristics between the two groups (Table S2, Supporting Information). Our genetic analysis demonstrated that EBV‐ NPC cases exhibited an overall higher rate of nonsynonymous mutations compared to EBV+ patients. This observation aligns with previous findings in other cancer types such as head and neck squamous cell carcinoma and gastric cancer, where virus‐negative cancers tend to have a higher nonsynonymous mutation rate than virus‐positive groups (Figure S1B, Supporting Information). These results highlight the distinct genetic landscapes and potential underlying mechanisms of EBV‐ NPC, contributing to our understanding of the disease and its association with viral status. Then, we conducted a detailed analysis of the mutation signature in both the EBV+ and EBV‐ groups.

To identify somatic mutation genes in EBV‐ nasopharyngeal carcinoma, we employed the CNVkit as an auxiliary detection method. We observed significant alterations in genes within the P53 pathway, with *TP53* variants detected in 59% of EBV‐ cases, which is higher than the mutation rate reported in EBV+ nasopharyngeal carcinoma. Moreover, other genes closely involved in *TP53* functionality, including *CDKN2A*, *MDM2*, *CREBBP*, and *ATM*, also exhibited mutations. *CDKN2A*, a cyclin‐dependent kinase inhibitor, plays a role in reducing *TP53* degradation through ubiquitination. Statistically, it is considered one of the most inactivated tumor suppressor gene in cancer. (Figure 1A and B). In contrast to previous reports, our data indicates that mutations in the NF‐κB pathway are not prominent. Earlier studies described a high frequency of mutations and deletions in *CYLD*, *TRAF3*, and *NFKBIA*, whereas in EBV‐ NPC, the rate of mutation and copy number variations (CNVs) of NF‐κB pathway related were lower than in previous studies (Figure 1A). Additionally, we observed significant CNVs in both *TGFBR2* and *MYC* in both patient groups. *TGFBR2* displayed notable deletions, while *MYC* showed significant amplification (Figure 1A and C). These findings suggest that EBV‐ and EBV+ NPCs might have both shared and also distinct genetic alterations and the functions of these alterations in NPC pathogenesis need further investigation.

### 2.2 An OPCM Strategy to Generate Primary, Orthotopic, and Genetically Defined NPC Models in Mice

**Figure 2:**
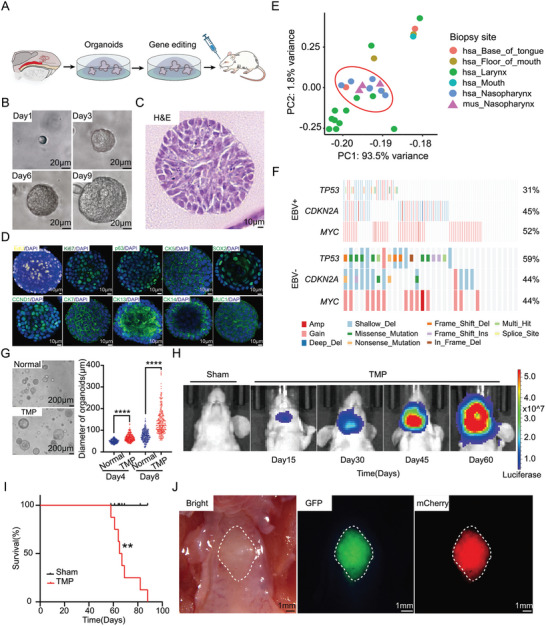
Generating primary and orthotopic NPC OPCMs with gene edited nasopharyngeal organoids in mice. A) Schematic of the strategy for generating primary nasopharyngeal cancer in nude mice with genome‐edited organoids. B) Microscopic images showing the time course of a monoclonal organoid generated from a single cell. Scale bar, 20 µm. C) Representative H&E image of normal nasopharyngeal organoids. Scale bar, 10 µm. D) Confocal images of organoids stained with EdU and labeled for a proliferative marker Ki67, basal epithelial cell markers p63 and CK5, stemness markers SOX2 and CCND1, a pseudostratified epithelial cell marker CK7, a stratified squamous epithelial cell marker CK13/CK14 and a goblet cell marker MUCI. Scale bar, 10 µm. E) The PCA analysis showing the similarity of mouse nasopharyngeal epithelium with human nasopharyngeal epithelium, compared to other head and neck epithelium. F) OncoPlot showing the SNPs and CNVs of *TP53*, *CDKN2A* and *MYC* in EBV+ and EBV‐ NPC patients. G) Representative images of normal nasopharyngeal organoids and engineered organoids with combinations of *Trp53*‐/‐; *Myc*; sg*Cdkn2a* (left). Scale bar, 200 µm. Comparison of sizes between normal nasopharyngeal organoids and engineered organoids with combinations of *Trp53*‐/‐; *Myc*; sg*Cdkn2a*, ****, *p* < 0.0001. Data are means ± SEM. Day 4 (n = 397); Day 8 (n = 340). H) Representative images of living images for primary and orthotopic NPC in nude mice. I) Survival curve of nude mice orthotopically transplanted with TMP premalignant organoids. n = 8 animals per group. **, *p* < 0.01. J) Representative macroscopic views of primary and orthotopic tumors in nude mice. Bright image of the upper jaw (left), GFP‐fluorescent primary and orthotopic tumors (middle), mCherry‐fluorescent primary and orthotopic tumors (right). Scale bar, 1 mm.

NPC presumably originates from the nasopharyngeal epithelium.^[^ ^26^ ^]^ The mouse nasopharyngeal tube epithelium is composed of pseudostratified ciliated columnar epithelium, stratified squamous epithelium, and transitional epithelium, similar to the human nasopharyngeal epithelium (Figure S2A, Supporting Information).^[^ ^27^, ^28^ ^]^ To bypass the various obstacles of traditional genetically engineered mouse models (GEMM), including lack of nasopharyngeal epithelium‐specific Cre, we developed a new strategy to generate primary and orthotopic NPC murine models with normal nasopharyngeal epithelial organoids with NPC‐associated genetic alterations by gene editing. We called these models as OPCM (Organoid‐initiated Precision Cancer Model), which has been successfully used for various types of cancers, including lung cancer,^[^ ^21^ ^]^ gastric cancer,^[^ ^22^ ^]^ bladder cancer,^[^ ^23^ ^]^ and endometrial carcinoma.^[^ ^24^ ^]^ First, we cultured organoids of nasopharyngeal epithelium from CAG‐Cas9‐EGFP; *Trp53‐/‐*; C57BL/6 mice (**Figure** **2**A). These 3D organoids, initiated from single epithelial cells, mainly displayed as solid spheres with smooth surfaces and grew rapidly (Figure 2B). Histological analyses showed that these organoids contained multiple layers of proliferating epithelial cells, indicated by EdU corporation assay and Ki67 staining. Then we performed whole mount staining to identify the cell types in these organoids. The results suggested that there were nasopharyngeal basal epithelial progenitor cells at the inner layers, indicated by the expressions of p63 and CK5, and stemness markers of SOX2 and CCND1. There were also multiple differentiated epithelial cells, including CK7 positive pseudostratified epithelial cells, CK13 and CK14 positive stratified squamous epithelial cells, and MUC1 positive goblet cells (Figure 2C and D). Further, we tested whether mouse nasopharyngeal organoids could represent the molecular features of human nasopharyngeal epithelium by transcriptomics analyses. We performed principal component analysis (PCA) of the transcriptomes of human nasopharyngeal epithelium and multiple other head and neck epithelium, including base of tongue, floor of mouth, larynx, and mouth, together with mouse nasopharyngeal epithelium. The result showed mouse nasopharyngeal epithelium were grouped together with its human counterpart, but not other head and neck epithelium, which strongly suggested that mouse nasopharyngeal epithelium were highly similar to human nasopharyngeal epithelium (Figure 2E). Additionally, we found both human nasopharyngeal epithelium and mouse nasopharyngeal organoids express typical markers of nasopharynx (Figure S2B, Supporting Information). Thus, mouse nasopharyngeal organoids could mimic both the cellular compositions and molecular features of the human nasopharyngeal epithelium.

We sought to generated mouse NPC models that represented patient‐relevant genetic alterations with engineered mutant nasopharyngeal organoids. Both published and our own data revealed that genetic alterations, including losses of *TP53* and *CDKN2A* (p16), and amplification of *MYC*, were highly recurrent in human NPC patients, indicating these alterations might promote NPC tumorigenesis (Figure 2F).

Then, we introduced NPC‐related genetic alterations into CAG‐Cas9‐EGFP; *Trp53‐/‐* nasopharyngeal organoids by mCherry‐linked sgRNA targeting *Cdkn2a* and overexpressing *Myc* together with luciferase, which could help to monitor these cells in vivo (Figure S2C and D, Supporting Information). T7E1 assay demonstrated the successful mutation of *Cdkn2a*, which was further confirmed by western blotting (Figure S2E and F, Supporting Information). QRT‐PCR assay and western blotting showed the overexpression of *Myc* in organoids (Figure S2G and H, Supporting Information). Compared to normal nasopharyngeal organoids derived from GAG‐Cas9‐EGFP C57BL/6 mice, CAG‐Cas9‐EGFP; *Trp53‐/‐*; *Myc*; sg*Cdkn2a* organoids (TMP) grew significantly faster (Figure 2G). In patients, all NPC developed in the nasopharynx and thus we tried to generate primary and orthotopic NPC by transplanting TMP nasopharyngeal organoids upon the pseudostratified columnar ciliated epithelium layer in the nasopharynx of nude mice (Figure S2I, Supporting Information). Living imaging showed that recipient mice of TMP organoids, but not those of control mice, displayed specific luciferase signaling in the nasopharynx site. And the luciferase signaling intensity dramatically increased over time, indicating the rapid growth of in situ tumors (Figure 2H; Figure S2J, Supporting Information). All of these recipients died of tumors about two months after transplantation (Figure 2I). There were obvious bulges at the upper palate with specific EGFP and mCherry expressions, indicating TMP organoids‐derived tumors (Figure 2J).

Taken together, we generated, as far as we know, the first primary, orthotopic, and genetically defined NPCs in mice with the OPCM strategy.

### 2.3 The Primary and Orthotopic Mouse NPC Recapitulates the Molecular and Clinical Characteristics of Human Disease

**Figure 3:**
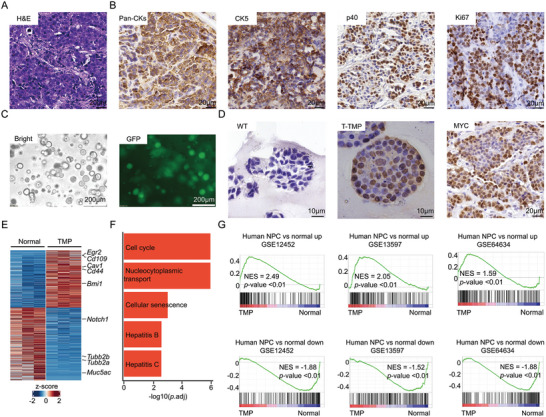
The primary and orthotopic mouse NPC OPCM recapitulates the molecular and clinical characteristic of human disease. A) Representative H&E staining of primary and orthotopic tumors. Scale bar, 20 µm. B) Representative IHC staining of primary and orthotopic tumors for Pan‐CKs, CK5, p40, and Ki67. Scale bar, 20 µm. C) Representative images of primary cultured organoids established from TMP primary and orthotopic tumors. Bright (left), GFP (right). Scale bar, 200 µm. D) Representative IHC staining pictures showing MYC levels in normal nasopharyngeal organoids (left), TMP tumor organoids (middle) and TMP tumor tissues (right). Scale bar, 10 µm. E) Heatmap showing the differentially expressed genes (*p*.adj < 0.05) in TMP tumor organoids versus normal nasopharyngeal organoids. F) Bar plots showing the top5 enriched KEGG pathways in TMP tumor organoids, comparing to those with normal nasopharyngeal organoids. G) GSEA showing enrichment of human NPC up‐ (top) and down‐regulated gene signatures (bottom) in the TMP tumor organoids, compared to normal nasopharyngeal organoids.

Next, we further investigated the histological and molecular features of the primary and orthotopic mouse NPC. Pathological analyses revealed that these tumor cells were less differentiated and highly mitogenic (**Figure** **3**A). Consistently, immunofluorescence staining showed that most of the tumor cells were Ki67 positive, indicating the severity of the disease. High levels of pan‐CKs, CK5, and p40 expressions suggested that they might be basal/squamous NPCs, which could recapitulate the histological features seen in human NPCs (Figure 3B).

To characterize the molecular features of these NPC models, we cultured tumor organoids derived from these in situ tumors and the GFP expression indicated that they were derived from the transplanted nasopharyngeal organoids. Histological analysis showed these tumor organoids maintained histologic features of their derived tissues (Figure 3C; Figure S3A, Supporting Information). T7E assay confirmed the disruption of *Cdkn2a* in mouse tumors, which was further confirmed by RNA sequencing (RNA‐seq) analyses (Figure S3B and C, Supporting Information). IHC staining showed the overexpression of *Myc* in the NPC organoids and tumors (Figure 3D). The transcriptomics analyses showed that the TMP tumor organoids had altered expressions of many NPC‐related genes, compared to normal nasopharyngeal organoids derived from Cas9‐EGFP C57BL/6 mice (Figure 3E). The KEGG cell cycle pathway was the top 1 upregulated in the TMP tumor organoids (Figure 3F). GSEA showed that the E2F target genes and the MYC target genes were significantly positively enriched (NES = 2.71, *p* < .001; NES = 2.32, *p* < .001, respectively) while the p53 pathway genes were significantly negatively enriched (NES = −1.93, *p* < .001) in tumor organoids compared to normal nasopharyngeal organoids, consistent with their genetic drivers of *Cdkn2a* deficiency, *Myc* overexpression and *Trp53* loss (Figure S3D, Supporting Information). Importantly, the upregulated genes in human NPC of all three independent studies were significantly positively enriched in the mouse NPC organoids (NES = 2.49, *p* < .001 for the Dodd gene signature;^[^ ^29^ ^]^ NES = 2.05, *p* < .001 for the Bose gene signature;^[^ ^30^ ^]^ and NES = 1.59, *p* < .001 for the Bo signature^[^ ^31^ ^]^). On the other side, the downregulated genes in all these human NPC were significantly negatively enriched in the mouse tumor organoids (NES = −1.88, *p* < .001 for the Dodd gene signature;^[^ ^29^ ^]^ NES = −1.52, *p* < .001 for the Bose gene signature;^[^ ^30^ ^]^ and NES = −1.88, *p* < .001 for the Bo gene signature^[^ ^31^ ^]^) (Figure 3G). Of note, GSEA revealed the TMP tumors had significantly upregulated expressions of undifferentiated cancer signature genes, which was associated with poor prognosis of NPC patients (Figure S3E, Supporting Information).

Distal metastases are a common feature of human NPC, especially those at the advanced stage. Presumably, NPC metastases into the lung and other frequent sites should migrate from the in situ primary tumors, which cannot be precisely represented in previous models. We checked distal metastases in our primary and orthotopic TMP NPC mice (Figure S3F, Supporting Information). H/E staining indicated that these lesions contained malignant epithelial cells, which also expressed CK5 and p63 and were Ki67 positive, similar to the primary NPC cells (Figure S3G, Supporting Information). Taken together, we generated primary and orthotopic NPC in mice, which recapitulated the pathology, molecular, and biological characteristics of human disease.

### 2.4 The Roles of *TP53* and *CDKN2A* Deficiency in NPC Tumorigenesis

**Figure 4:**
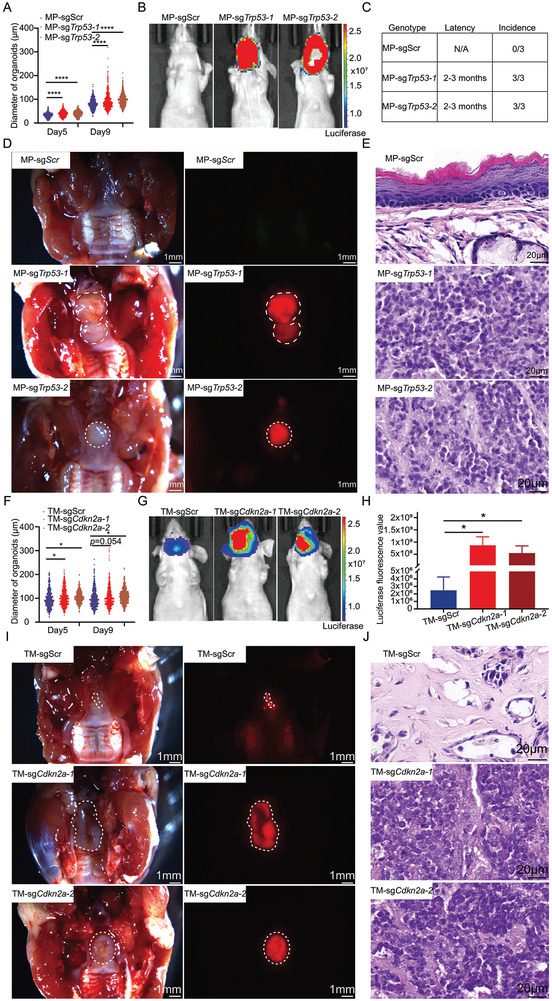
*Trp53* and *Cdkn2a* loss play pivotal roles in NPC pathogenesis. A) Comparison of sizes between engineered MP‐sgScr, MP‐sg*Trp53‐1* and MP‐sg*Trp53‐2* premalignant organoids. ****, *p* < 0.0001. Data are means ± SEM. Day 5 (MP‐sgScr (n = 403), MP‐sg*Trp53‐1* (n = 426), MP‐sg*Trp53‐2* (n = 401)); Day 9 (MP‐sgScr (n = 623), MP‐sg*Trp53‐1* (n = 662), MP‐sg*Trp53‐2* (n = 671)). B) Representative images of living images for nude mice transplanted with MP‐sgScr, MP‐sg*Trp53‐1*, MP‐sg*Trp53‐2* premalignant organoids. C) Situation of nude mice orthotopically transplanted with MP‐sgScr, MP‐sg*Trp53‐1*, and MP‐sg*Trp53‐2* premalignant organoids. D) Representative macroscopic views of primary and orthotopic tumors. Bright image of the upper jaw (left), mCherry‐fluorescent primary and orthotopic tumors (right). Scale bar, 1 mm. E) Representative H&E staining of primary and orthotopic MP and TMP tumors. Scale bar, 20 µm. F) Comparison of sizes between engineered TM‐sgScr, TM‐sg*Cdkn2a‐1* and TM‐sg*Cdkn2a‐2* premalignant organoids. *, *p* < 0.05. Data are means ± SEM. Day 5 (TM‐sgScr (n = 601), TM‐sgCdkn2a‐1 (n = 621), TM‐sgCdkn2a‐2 (n = 600)); Day 9 (TM‐sgScr (n = 657), TM‐sgCdkn2a‐1 (n = 662), TM‐sgCdkn2a‐2 (n = 600)). G) Representative images of living images for nude mice transplanted with TM‐sgScr, TM‐sg*Cdkn2a‐1*, TM‐sg*Cdkn2a‐2* premalignant organoids. H) Comparison of luciferase values of mice transplanted with TM‐sgScr, TM‐sg*Cdkn2a‐1*, TM‐sg*Cdkn2a‐2* premalignant organoids. n = 3 animals per group. *, *p*<0.05. I) Representative macroscopic views of primary and orthotopic tumors. Bright image of the upper jaw (left), mCherry‐fluorescent primary and orthotopic tumors (right). Scale 1 mm. J) Representative H&E staining of primary and orthotopic TM and TMP tumors. Scale bar, 20 µm.

The genetic landscape of NPC indicated that mutations or deletions of *TP53* and *CDKN2A* might play pivotal roles in NPC pathogenesis (Figure 1A). However, the biological functions of these genes during the progression of NPC remains poorly defined. Hence, we wanted to define the roles of *TP53* and *CDKN2A* deficiency in NPC tumorigenesis based on our NPC OPCM models. First, we generated genetic defined *Myc*; sg*Cdkn2a* (MP) nasopharyngeal organoids with (MP‐sg*Trp53*) or without (MP‐sgScr) disrupting *Trp53* by gene editing (Figure S4A, Supporting Information). Organoids with mutation of *Trp53* grew significantly faster than its control counterpart (**Figure** **4**A; Figure S4B and C, Supporting Information). Then, we transplanted premalignant MP and TMP organoids into the nasopharynx site of recipient mice. Living imaging showed that recipient mice transplanted with TMP organoids, but not those of control mice, displayed specific luciferase signaling in the nasopharynx site (Figure 4B). No tumors were observed in recipient mice transplanted with MP organoids, and TMP organoids transformed into tumors following a 2–3‐months latency in 3/3 of mice (Figure 4C–E). These results indicated that mutation of *Trp53* was a crucial event for NPC initiation. On the other hand, we also tested biological function of mutation of *Cdkn2a* during NPC tumorigenesis. Similarly, we found organoids with deficiency of *Cdkn2a* grew significantly faster than its control counterpart (Figure 4F; Figure S4D–F, Supporting Information). Of note, living imaging showed that recipient mice transplanted with organoids with genetic combinations of *Trp53*‐/‐; *Myc*; sgScr (TM) and *Trp53*‐/‐; *Myc*; sg*Cdkn2a* (TMP) all displayed specific luciferase signaling in the nasopharynx site (Figure 4G). However, the value of luciferase signaling of TMP mice were significantly higher than TM mice (Figure 4H). Despite both TM and TMP mice gave rise to mCherry‐specific tissues, pathological analyses indicated that TM organoids could not transform into tumors. Unlike TMP tumors with histological feature of low‐/un‐ differentiated cancer, TM tissues displayed epithelium dysplasia (Figure 4I,J). These results suggested *Cdkn2a* loss promoted transformation of cancer cells. Taken together, these results indicated mutations or deletions of *TP53* and *CDKN2A* were important for NPC initiation and progression.

### 2.5 LMP1 Enhances the Distal Metastasis of NPC

**Figure 5:**
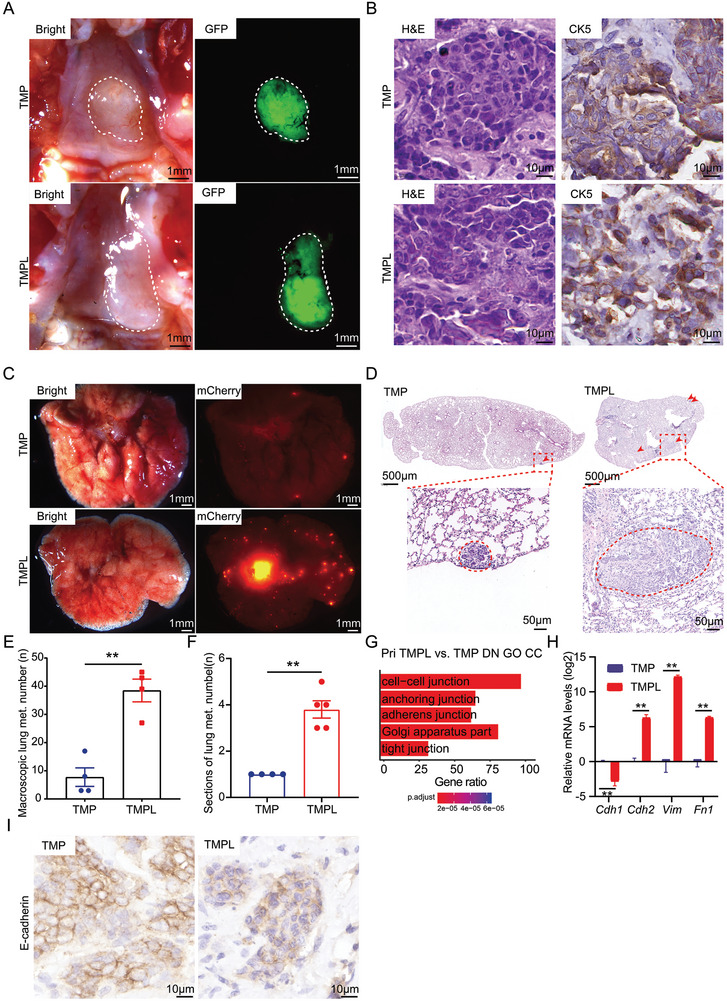
LMP1 promotes distal metastasis of NPC. A) Representative macroscopic views of primary and orthotopic tumors generated from TMP and TMPL premalignant organoids. B) Representative H&E staining of primary and orthotopic TMP and TMPL tumors (left). Representative IHC staining of primary and orthotopic TMP and TMPL tumors for CK5 (right). Scale bar, 10 µm. C) Representative images of macroscopic lung micrometastases. bright images of lung lobes (left). mCherry‐fluorescent metastasized tumors in lung lobes (right). Scale bar, 1 mm. D) The representative H&E staining of the lung section of TMP and TMPL NPC mice. Scale bar, 50 µm. E) Comparison of the numbers of macroscopic lung metastases between TMP and TMPL mice (left). n = 4 animals per group. **, *p* < 0.01. F) Comparison of the number of metastatic lung lesions in lung sections of TMP and TMPL NPC mice. n = 4 sections for TMP tumors, n = 5 sections for TMPL tumors. **, *p* < 0.01. G) Bar plots showing the most enriched DN GO CC pathways in TMPL primary tumors, compared to TMP primary tumors. H) Analysis of relative expression levels of EMT marker genes in TMP and TMPL primary tumors using QRT‐PCR. Data shows the means ± SEM (n = 3). **, *p* < 0.01. I) Immunohistochemical analysis of expression of E‐cadherin in TMP and TMPL primary tumors. Scale bar, 10 µm.

EBV infection is strongly associated with NPCs, but EBV is a human‐specific virus and can't infect murine epithelial cells.^[^ ^32^, ^33^, ^34^ ^]^ Given that there is accumulating evidence suggesting that LMP1 would be an important viral oncogene of EBV in NPC and other cancers,^[^ ^35^, ^36^, ^37^, ^38^, ^39^, ^40^, ^41^, ^42^, ^43^ ^]^ we wondered if LMP1 would play significant roles in NPC initiation and progression in our murine models and at least partially mimic the functions of EBV in patients (Figure S5A, Supporting Information). We introduced LMP1 into normal nasopharyngeal organoids and, intriguingly, organoids with LMP1 grew significantly slower than those with empty vector, probably due to the cellular toxicity of abundant LMP1 as previously reported^[^ ^44^, ^45^ ^]^ (Figure S5B and C, Supporting Information). Then, we tested the potential in vivo functions of LMP1 in NPC tumorigenesis by introducing it into premalignant TMP nasopharyngeal organoids (TMPL). The expression of LMP1 was validated by QRT‐PCR and western blotting (Figure S5D and E, Supporting Information). However, paradoxically, once transplanted into the nasopharynx of recipient nude mice, TMPL organoids exhibited comparable tumorigenesis capability as TMP, demonstrated by the disease latency (**Figure** **5**A; Figure S5F, Supporting Information). The TMPL tumors displayed similar histology and marker genes expressions to the TMP tumors (Figure 5B). Similar to the TMP tumors, the TMPL NPC also expressed p63 and Ki67, indicating aggressive squamous epithelial malignancies (Figure S5G, Supporting Information). Infiltration of CD20^+^ B cells in TMP and TMPL tumors were visualized by IHC staining (Figure S5H, Supporting Information). It has been shown that EBV and LMP1 itself could disrupt the immune response of host cells for its immune escape and consistently, transcriptomes of the TMP and TMPL tumors showed that many immune response pathways, including HALLMARK INTERFERON ALPHA RESPONSE (NES = −2.33, *p* = 0.00), HALLMARK INTERFERON GAMMMA RESPONSE (NES = −1.75, *p* = 0.00), and GO POSITIVE REGULATION OF CYTOKINE PRODUCTION IN IMMUNE RESPONSE (NES = −1.68, *p* = 0.01), were significantly negatively enriched in the TMPL NPC compared to the TMP tumors (Figure S5I and J, Supporting Information). These results demonstrated the functions of LMP1 in NPC tumorigenesis and further confirmed it as a key oncogene of EBV in NPC.

Of note, despite the similarity of the primary tumors, we found that the mCherry positive loci in the lung of TMPL recipients were about a five‐fold increase to those of the TMP recipients. Pathological analyses confirmed there were significantly more metastases of TMPL tumors than those of TMP tumors (Figure 5C–F). These results strongly supported the metastasis‐promoting function of LMP1 in the primary and orthotopic NPCs.

To characterize the tumor‐intrinsic molecular rewiring associated with its enhanced metastasis by LMP1 in NPC, we cultured NPC organoids from the primary tumors and lung metastases with or without LMP1. Interestingly, while most of the tumor organoids from the lung metastases without LMP1 were hollow, all of the ones with LMP1 were solid after a long‐time culture (Figure S5K, Supporting Information). By transcriptome analyses, GSEA showed that gene signatures associated with invasion in multiple human cancers were significantly enriched in the TMPL primary NPC organoids, compared to the TMP primary NPC organoids (Figure S5L and M, Supporting Information). Epithelial‐to‐mesenchymal transition (EMT) is one of the most common mechanisms underlying metastases. Of note, GSEA revealed that the EMT gene signature was significantly positively enriched in the TMPL NPC compared to the TMP tumors (NES = 1.28, *p* < .001) (Figure S5N, Supporting Information). Consistently, we found that multiple cell‐cell interactions GO pathways were downregulated by LMP1 in the primary NPC (Figure 5G). QRT‐PCR assays confirmed that the epithelial gene *Cdh1* was significantly downregulated while the mesenchymal genes, *Cdh2, Vim* and *Fn1*, were significantly upregulated in the TMPL tumor organoids compared to the TMP organoids (Figure 5H). Immunohistochemistry staining validated that the protein level of *Cdh1* (*E‐cadherin*) was decreased in the TMPL NPC (Figure 5I). These data suggested that LMP1 might promoted NPC metastasis through the EMT reprogramming.

### 2.6 *TGFBR2* Deficiency Promotes Malignancy of NPC

**Figure 6:**
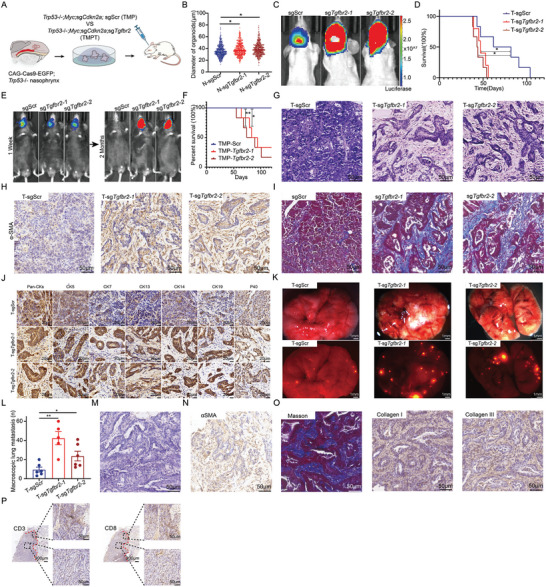
*Tgfbr2* inactivation accelerates malignancy of NPC. A) Schematic diagram of the strategy for exploring functions of *Tgfbr2* loss with premalignant gene‐edited organoids in nude mice. B) Comparison of sizes between TMP premalignant organoids and TMPT premalignant organoids, *, *p* < 0.05. Data are means ± SEM, N‐sgScr(n = 502), N‐sg*Tgfbr2‐1*(n = 511), N‐sg*Tgfbr2‐2*(n = 511). C) Representative images of living images for nude mice transplanted with TMP‐sgScr, TMP‐sgScr, TMP‐sg*Tgfbr2‐1*, and TMP‐sg*Tgfbr2‐2* premalignant organoids. D) Survival curve of nude mice orthotopically transplanted with TMP and TMPT premalignant organoids. T‐sgScr (n = 6); T‐sg*Tgfbr2‐1* (n = 5); T‐sg*Tgfbr2‐2* (n = 6). *, *p* < 0.05. E) Representative images of living images for C57BL/6 mice transplanted with tumor organoids derived from TMP and TMPT primary tumors. F) Survival curve of C57BL/6 mice orthotopically transplanted with TMP and TMPT tumor organoids. n = 6 animals per group. *, *p* < 0.05, **, *p* < 0.01. G) Representative H&E staining of primary and orthotopic tumors of TMP and TMPT mice. Scale bar, 50 µm. H) Representative IHC staining of TMP and TMPT primary tumors for αSMA. Scale bar, 50 µm. I) Masson staining of TMP and TMPT primary tumor tissues. Collagens (blue). Cytoplasm (Red). Scale bar, 50 µm. () Representative IHC staining of TMP and TMPT primary tumors for Krts and p40. Scale bar, 20 µm. K) Representative images of macroscopic lung metastasized tumors of TMP, TMP‐sg*Tgfbr2‐1*, and TMP‐sg*Tgfbr2‐2*. bright image of lung lobes (up). mCherry‐fluorescent metastasized tumors in lung lobes (down). Scale bar, 1 mm. L) Comparison of the numbers of macroscopic lung metastases between TMP and TMPT mice. T‐sgScr (n = 6); T‐sg*Tgfbr2‐1* (n = 5); T‐sg*Tgfbr2‐2* (n = 6). *, *p* < 0.05, **, *p* < 0.01. M) Representative H&E staining of TMPT tumors in C57BL/6 mice generated by second transplantation of tumor cells in nude mice. Scale bar, 50 µm. N) Representative IHC staining TMPT tumors for αSMA in C57BL/6. Scale bar, 50 µm. O) Masson staining of TMPT tumors in C57BL/6 mice. Collagens (blue). Cytoplasm (Red). Scale bar, 50 µm (Left). Representative IHC staining of TMPT tumors for collagen I and collagen III in C57BL/6 mice. Scale bar, 50 µm (Middle, and Right). P) Representative IHC staining of TMPT tumors for CD3 and CD8 in C57BL/6 mice. Scale bar, 50 µm.

A notable advantage of OPCM, comparing to GEMM, is very convenient to study any given cancer‐associated gene mutation or combination of mutations in tumorigenesis. As an example, *TGFBR2*, a constituent part of TGFβ receptor, is frequently mutated or deleted in both EBV+ and EBV‐ NPC patients, in NPC initiation and progression (Figure 1A). Consistent with previous reports, we confirmed the downregulation of TGFBR2 protein levels in the clinical NPC samples, compared to its counterparts of normal human tissues (Figure S6A and B, Supporting Information).^[^ ^46^ ^]^ Low expressions of *TGFBR2* were associated with advanced disease and poor prognosis of NPC patients (Figure S6C and D, Supporting Information).^[^ ^47^ ^]^ To intuitively explore the biological function of mutation of *TGFBR2* during the progression of NPC, we chose to introduce CRISPR‐Cas9‐mediated inactivation of *Tgfbr2* in the engineered mutant TMP premalignant organoids (TMPT) (**Figure** **6**A; Figure S6E, Supporting Information). Western blotting analysis confirmed decreased level of *Tgfbr2* protein and disrupting TGF‐β/SMAD signaling, including reduced‐expression of p‐SMAD2/3 (phosphorylated SMAD2/3), SMAD4, and increased‐expression of SMAD2/3, in *Tgfbr2* mutant organoids. The reduced‐expression of p‐SMAD2/3 was consistent with well‐known function of TGFβ receptor (Figure S6F, Supporting Information). Additionally, comparing to *Tgfbr2*‐wildtype nasopharyngeal premalignant organoids, *Tgfbr2* mutant premalignant organoids grew significantly faster (Figure 6B; Figure S6G, Supporting Information). Then, we injected premalignant TMP organoids with or without mutation of *Tgfbr2* into the nasopharynx of mice. Comparing to TMP tumors, TMPT tumors grew significantly faster (Figure 6C; Figure S6H and I, Supporting Information). And *Tgfbr2* mutation significantly accelerated NPC progressing and reduced the overall survival of receptor mice (Figure 6D). Consistently, tumors‐derived TMPT organoids similarly grew significantly faster than tumors‐derived TMP tumor organoids (Figure S6J and K, Supporting Information). GSEA analysis showed that “HALLMARK TGF BETA SIGNALING” pathway was significantly negatively enriched in the TMPT tumors compared to the TMP tumors, consist with deficiency of *Tgfbr2* (Figure S6L, Supporting Information). Moreover, we orthotopically transplanted tumor organoids derived from TMP and TMPT nude mice into immune complete C57BL/6 mice to investigate the role of *Tgfbr2* loss in immune evasion. Interestingly, we found only the TMPT tumor organoids, not TMP tumor organoids eventually developed tumors in C57BL/6 recipients (Figure 6E and F; Figure S6M and N, Supporting Information). The result suggested *Tgfbr2* loss promoted malignancy of NPC and protected cancel cells from cytotoxicity T lymphocytes. Interestingly, histological analysis showed that TMPT tumors exhibited a significant increase of stromal areas as compared to TMP tumors (Figure 6G; Figure S6O, Supporting Information). We further found the ratio of stromal volume with the number of αSMA‐expressing fibroblasts were significantly increased in TMPT tumors comparing with those in stromal areas of TMP tumors (Figure 6H). Considering fibroblasts were dominant source of collagens, then, increased collagens detected by both Masson staining and IHC staining were consistently found in TMPT stomal areas comparing with stromal areas in TMP tumors (Figure 6I; Figure S6P, Supporting Information). These results indicated that cancer cells‐intrinsic *Tgfbr2* loss promoted desmoplastic reaction. Additionally, we performed IHC staining of TMP and TMPT tumors for cytokeratin family markers and squamous cancer markers of p40. The results indicated the proteins levels of cytokeratin family markers and squamous cancer markers of p40 were significantly increased in TMPT cancer cells, comparing to TMP cancer cells (Figure 6J). Importantly, we found there were significantly increased mCherry positive loci in the lung of TMPT recipients, comparing to TMP recipients (Figure 6K,L). Histological analysis further confirmed TMPT mice exhibited significantly increased metastases in lung lobes, suggesting considerable potency of *Tgfbr2* mutation in driving NPC metastasis (Figure S6Q and R, Supporting Information).

Then, we further explored the mechanism of immune evasion driving by *Tgfbr2* loss in immune complete C57BL/6 mice. Transcriptomes analysis of TMP and TMPT tumors derived from nude recipient mice showed that many immune response pathways were significantly downregulation in TMPT tumors compared to TMP tumors (Figure S6S, Supporting Information). This result suggested that *Tgfbr2* loss might repress the immune response and thus help the tumor cells for their immune escape. Besides, pathological analysis showed that TMPT tumors in C57BL/6 mice enriched fibroblasts and collagens in stomal areas (Figure 6M–O). We further analyzed infiltration of T cells within the TMPT NPC models in C57BL/6 mice. And we found T cells were enriched in junction areas of TMPT tumor and normal tissues, but excluded in intratumor regions, suggesting *Tgfbr2* loss promoted immune evasion through excluding T lymphocytes infiltration (Figure 6P). Importantly, we found there were consistently less infiltration of macrophages, B cells, and T cells in *Tgfbr2* low expression NPC patients compared with *Tgfbr2* high expression NPC patients (Figure S6T, Supporting Information). Taking together, these results suggested *Tgfbr2* loss promoted tumorigenesis of NPC and promoted immune evasion of NPC through concerting tumor microenvironment (TME) to a fibroblasts‐enriched, collagens‐filled, and lymphocytes excluded state.

## 3 Discussions

In this study, we created a serial of primary, orthotopic, and genetic driver‐defined NPC mouse models initiated with gene‐edited normal nasopharyngeal organoids. We called this type of cancer models as Organoid‐initiated Precision Cancer Models (OPCM), which we and other laboratories have applied for several other types of cancers, including lung cancer,^[^ ^21^ ^]^ gastric cancer,^[^ ^22^ ^]^ endometrial cancer,^[^ ^24^ ^]^ and colorectal cancer.^[^ ^48^, ^49^, ^50^ ^]^ Traditionally genetically engineered mouse models (GEMMs) have been the gold standard in modeling various human cancers because they were initiated with defined genetic onco‐drivers and in most cases developed in situ with the proper microenvironment.^[^ ^51^, ^52^ ^]^ While maintaining these advantages of GEMM, OPCM is more convenient to introduce any genetic alteration or combination of multiple genetic alterations. Due to many reasons, including lack of tissue‐specific Cre and unclear initiating cell identities, GEMMs for some types of cancers are limited or unavailable and, in contrast, OPCM can be universally used for all types of cancers. With this strategy, here, we report, as far as we have known, the first primary and orthotopic NPC models. the new NPC models faithfully recapitulate the pathological and molecular features of human disease. In contrast to most of the previous NPC models, these new models represent the whole process of NPC tumorigenesis.^[^ ^53^ ^]^ Therefore, they would be essential to study the cellular and molecular events during different stages of NPC tumorigenesis. As we have demonstrated here, the potential biological functions of NPC‐associated genetic alterations could be investigated in these models. And further, these models would also be useful for preclinical tests of potential therapeutic targets and treatment, including immunotherapies, given their recapitulation of the pathogenesis.

NPC is a heterogenous disease in term of pathogenesis and clinic outcomes. Despite more than 90% NPCs are associated with EBV infection, there are some NPC patients without EBV infection. By comparing their genomics, we find that ENV‐ NPCs seem have higher tumor mutation burdens, which is consistent with the proposed oncogenic roles of EBV. However, EBV+ and EBV‐ NPCs also displayed many common altered genes, such as amplification of *MYC*, Loss of *TP53*, and mutation of *CDKN2A*, suggesting that these genes would be critical for NPC pathogenesis. With our OPCM of NPC, we experimentally validated *TP53* and *CDKN2A* as bona fide tumor suppressors of NPC. Intriguingly, *TP53* and *CDKN2A* seem to have different roles in the initiation and progression, respectively, of NPC tumorigenesis. The shared and distinct pathogenesis of EBV+ and EBV‐ NPC need further studies.

Distal metastasis is the major lethal cause of NPC patients, but its molecular mechanisms remain unclear.^[^ ^54^ ^]^ Here, with the OPCMs of NPC which displayed distal metastasis from the primary tumors, we reveal multiple mechanisms of NPC lung metastasis. We find that LMP1, a key putative oncogenic gene of EBV and toxic to normal nasopharyngeal epithelium, seems to have little effect on primary tumors but dramatically promote its lung metastasis, probably through EMP reprogramming. Of note, EBV has been shown to be tumorigenic in NPC possibly due to expressing viral oncogenes latent membrane proteins (LMP) and EBV‐determined nuclear antigens (EBNA). However, because EBV is endemic to human, it is impossible to directly study EBV infection in mice. EBV promotes NPC by regulating the gene expressions and pathways of the host genome through its encoded oncogenic proteins. Therefore, our study and multiple other studies have modeled EBV‐induced tumorigenesis in mice by expressing EBV genes, especially LMP1, into mouse cells. And although our murine models are suitable for exploring functions of EBV oncogenic proteins in NPC initiation and progression and at least partially mimic the functions of EBV in patients, due to the lack of the complete virus, it is difficult to explore the oncogenic functions of complete EBV or interaction of various EBV oncogenic proteins during NPC tumorigenesis.

Further, we discovered that loss of *TGFBR2*, frequently mutated in NPC and other types of human cancers, not only promoted the progression of NPC in mice, but also the lung metastasis. *TGFBR2* has been shown to be frequently mutated, deleted, or repressed in NPC and associated with poor prognosis. The TGF beta pathway is a master regulator of cancer metastasis. Our findings are consistent with previous reports on the roles of *TGFBR2* in other types of cancers, such as breast cancer,^[^ ^55^ ^]^ colorectal cancer,^[^ ^56^, ^57^ ^]^ and lung cancer.^[^ ^58^ ^]^ In our mouse OPCM, we show that *Tgfbr2* deficiency might promote metastasis through reprogramming the tumor microenvironment, especially increased cancer‐associated fibroblasts. However, the molecular mechanism of cancer cells‐intrinsic *Tgfbr2* loss promoting desmoplastic reaction in NPC is unclear. Dhainaut, M., et al.^[^ ^58^ ^]^ observed similarly increased infiltration of fibroblast in lung cancer due to TGFβ‐receptor loss on cancer cells increased TGFβ bioavailability effects on the TME. There may be a similar molecular mechanism of *TGFBR2* loss to reprogram TME in NPC and lung cancer. On the other hand, *Tgfbr2*‐deficient tumor cells may also alter the tumor microenvironment by increasing the secretion of fibroblasts‐recruiting factors. These observations need to be confirmed in patients and the detailed molecular mechanisms need to be further investigated.

## 4 Experimental Section

### NPC Tissue Collection

A total of 32 tissue samples from EBV‐ nasopharyngeal carcinoma patients were obtained for whole exome sequencing, with tumors or healthy controls sourced from the Sun Yat‐sen University Cancer Center biobank. This study was approved by the Institutional Review Board of the Sun Yat‐sen University Cancer Center (approval number B2023‐316‐01). Informed consent was obtained from all study participants.

### Mice

All mice experiments were performed in the State Key Laboratory of Biotherapy of Sichuan University and approved by the Institutional Animal Care and Use Committees of Sichuan University. Nasopharyngeal epithelial cells were obtained from the nasopharyngeal tube of C57BL/6 (Jackson Lab, Cat# 000664) and *Trp53‐/‐*; CAG‐Cas9‐EGFP mice (Jackson Lab, Cat# 02 6179). For orthotopic transplantation, the engineered organoids were orthotopically injected into the nasopharynx position of immunodeficient Nude mice (Hfkbio, Cat# BALB/cA‐nu Mice) and C5BL/6 mice at a young age (6–8 weeks, male) using insulin syringes. All mice were randomly grouped before transplantation. The tumor monitoring process was performed using a whole‐body image system.

### Plasmids Construction

The plasmids for expression of *Myc* were constructed into retroviral constructs including MSCV‐*Myc‐*IRES‐Luci2. The plasmid for expression of LMP1 was constructed into retroviral constructs including MSCV‐LMP1‐EFS‐BFP. The construct for sgRNAs (Table S3, Supporting Information) were designed on the CRISPR Design tool (http://crispr.mit.edu/) and were constructed into lentiviral constructs including MSCV‐ sgRNA‐ EFS‐ mCherry. The primers for T7 analyses were designed on Integrated DNA technologies (http://sg.idtdna.com/). Primers used for T7 analyses were shown in Table S4 (Supporting Information). Lentivirus packaging and cell infection were implemented as described.^[^ ^59^ ^]^

### Cell Lines

293T, 3T3 cell lines were obtained from American Type Culture Collection (ATCC). All cell lines were maintained in DMEM medium supplemented with 10% fetal bovine serum, and penicillin (100 U mL^−1^)/streptomycin (0.1 mg mL^−1^) at 37 °C with 5% CO_2_. All cell lines cultures were regularly tested for mycoplasma contamination using PCR.

### Organoid Culture

Normal nasopharyngeal tissue or tumors were prepared for organoid culture. The isolated tissues were digested into single cells with 1×0.25% Trypsin‐EDTA (GIBCO, Cat# 2 152 925) for 1 h at 37 °C water bath and embedded in 30 µl 3D Matrigel matrix (BD, Cat# 354 230), supplemented with 150 µl full medium containing 1 x B27 (GIBCO, Cat# A3582801), 1 x N2 (GIBCO, Cat# 17 502 048), EGF (R&D, final 50 ng mL^−1^), FGF10 (Peprotech, Cat# 100‐26‐1000, final 200 ng mL^−1^), A27632 (Abmole Bioscience, final 10 µM),A83‐01 (Peprotech, Cat# 9 094 360, final 2 µm), R‐spodin 1 (Peprotech, Cat# 120‐38‐1000, final 250 ng mL^−1^), Noggin (Peprotech, Cat# 120‐10C‐250, final 100 ng mL^−1^), 10% Wnt‐3A conditioned medium, Nicotinamide (Sigma, Cat # N0636, 1 mM), N‐acetylcysteine (Sigma, Cat# A9165, 1 mM), Glutamax (Peprotech, Cat# 35050–061, 2 mM), and 1 x Penicillin/Streptomycin (GIBCO, Cat# 15140‐122) in DMEM‐F12 (Cat# 8 121 062). Organoids were maintained at 37 °C with 5% CO_2_ in an incubator.150 µl fresh medium was added to the dish to avoid drying up of the culture after 3 days. For maintenance, the established organoids were dissociated into single cells with TrypLE (GIBCO, Cat# 12605‐028), and passaged at 1:2 or 1:3 ratio. Pictures of organoids were obtained by using an inverted fluorescence microscope (OLYMPUS).

### Organoid Genome Editing

The constructs for sgRNAs were designed with the CRISPR Design Tool (http://crispr.mit.edu/) and cloned into lentiviral constructs including MSCV‐ sgRNA‐ EFS‐ mCherry (Table S3, Supporting Information). The cultured organoids were dissociated into single cells with TrypLE and resuspended with lentiviral supernatants supplemented with 1: 1000 (v/v) polybrene and centrifuged at 800 g for 1 h followed by 2 h incubating. The processed organoids were resuspended with Matrigel and seeded in plates for continue culturing. The mutations of sgRNAs targeting desired genes were confirmed by T7 restriction endonuclease analysis. The primers for T7 analyses were designed on Integrated DNA technologies (http://sg.idtdna.com/). Primers used for T7 analyses were shown in Table S4 (Supporting Information).

### Organoid Orthotopic Transplantation

The expanded organoids were dissociated from the Matrigel matrix using TrypLE at 37 °C for 3 min. Then the cells were collected by centrifugation for 5 min at 400 g, and resuspended in Matrigel placed on ice. For orthotopic transplantation, the recipient mice were deeply anesthetized with isoflurane and placed in a supine position. The mouth of mice was held open with the tongue pulled aside to completely expose the hard palate and ventral surface of the soft palate. Then, 100000 cells were resuspended in 10 µl Matrigel were rapidly injected into the nasopharynx of nude mice or C57BL/6 recipient mice using insulin syringes. Paired 10 µl organoids‐free Matrigel was used as a negative control. For exploring functions of *Trp53*, *Cdkn2a*, *Tgfbr2* and LMP1 underlying tumorigenesis, the same number of cells into the recipient mice. 100000 cells resuspended in 10 µl Matrigel were injected into the nasopharynx of the recipient mice using insulin syringes. The whole process of orthotopic injection was performed under a microscope in a sterile condition.

### Bioluminescent Imaging

For bioluminescent imaging, mice were injected with 150 mg kg^−1^ D‐luciferin potassium salt (Biovision, Cat# 7903–10PK) intraperitoneally and imaged on the IVIS Spectrum In Vivo Imaging System (PerkinElmer).

### Western Blotting

The proteins for western blotting analysis were extracted with RIPA buffer (Beyotime, Cat#P0013) supplemented with protease inhibitors (Beyotime, Cat#P1045). Then, the proteins were resolved SDS–PAGE gel electrophoresis and transferred to PVDF membranes. Followed by incubating with primary antibodies (Table S5, Supporting Information) against MYC, CDKN2A, LMP1, p‐SMAD2/3, SMAD2/3, SMAD4, and TGFBR2, diluted in TBST buffer with 5% non‐fatty milk at 4 °C overnight. Horseradish peroxidase (HRP)‐conjugated secondary antibodies were applied, and incubated for 1 h at room temperature. NcmECL Ultra Reagent (NCM biotech) was using for imaging.

### Whole‐Mount Staining

Fresh organoids embedded in Matrigel were fixed with 4% paraformaldehyde/PBS for 30 min at room temperature. Organoids were permeabilized with permeabilization solution (PBS containing 0.5% Triton X‐100) for 30 mins, blocked with 1% bovine serum albumin (BioFROXX, Cat# EZ6789A164) for 1 h, and incubated with primary antibodies (Table S5, Supporting Information), diluted in PBS with 0.05% Triton X‐100, 0.2% Tween and 1% BSA at 4 °C overnight. Followed by incubating with secondary antibodies Coupled by Alexa Fluor 488 (Abcam, ab150117), cy3(Jackson ImmunoResearch, Cat#146 340), and Alexa Fluor 647 (Jackson ImmunoResearch, Cat#144 067), respectively. Confocal images of organoids were obtained using a ZEISS LSM 800 confocal microscope.

### Histology and Immunostaining

Tumor tissues were fixed with 4% PFA, embedded in paraffin, and cut into 5‐µm‐thick sections. For hematoxylin‐eosin Staining, slices were stained with hematoxylin and eosin according to standard protocol (Servicebio, Cat# G1005). For Masson staining, slices were stained reagents according to standard protocol (Solarbio, Lot.No. 20 220 330). For immunohistochemistry, antigen retrieval was performed with 10 mM sodium citrate buffer after deparaffinization and rehydration. After permeabilization with 0.3% Triton‐X and blocking with 2% goat serum, slices were incubated with primary antibodies (Table S5, Supporting Information) at 4 °C overnight. Horseradish peroxidase (HRP)‐conjugated secondary antibodies (ZSGB‐BIO, Cat# 2127A0609) were applied and incubated for 1 h at room temperature. Then, DAB (ZSGB‐BIO, Cat# ZLI‐9017) was applied to envision, and hematoxylin for nuclear staining. The staining images were scanned with a panoramic MIDI (3DHISTECH).

### RT‐qPCR

For RT‐qPCR, total RNA was extracted from organoids with TRIzol (Applied Biosystems, Cat # 15 596 026). cDNA was synthesized with the cDNA Synthesis Kit (Vazyse, Cat# R323‐01‐AB) following the manufacturer's protocol. qPCR was performed with SYBR Green PCR Master Mix (Applied Biosystems, Cat# A25741). All reactions were repeated in three independent experiments with three technical repetitions for each sample. Primers used for qPCR analysis were shown in Table S6 (Supporting Information).

### In situ Hybridization

Tumor tissues were fixed with 4% PFA, embedded in paraffin, and cut into 3‐µm‐thick sections. To detect EBERs, the EBER detection kit (ISH‐7001; OriGene Technologies, Inc.) was used and followed the manufacturer's instructions: After dewaxing the paraffin sections, blocking solution was added and incubated at room temperature in the dark, followed by the addition of pepsin working solution and incubation at 37 °C for 30 min. After washing, 10 uL of digoxigenin‐labeled EBER probe was added and hybridization was performed at 37 °C for 4 h. After washing, 50 uL of HRP‐labeled anti‐digoxigenin antibody was added and incubated at 37 °C for 30 min. After washing with PBS buffer, DAB coloring solution was added and incubated at room temperature for 5 min. The slides were then restained with hematoxylin for 5 s and coverslipped.

### Whole Exome Sequencing

For each sample, 40–200 ng of genomic DNA was collected, which was then fragmented into 200–300 bp fragments using the Covaris ultrasonication system. Subsequently, it was performed end repair, 5′ phosphorylation, and added barcoded sequences to these fragmented DNAs. All these steps were performed using the SPARK Lib Prep Kit, following the manufacturer's instructions for DNA library preparation. The exon regions were captured using the SureSelect V6 whole exon kit from Agilent for all samples, and all the kits were within their expiration dates. Subsequently, it was performed sequencing using the Illumina HiSeq 2500 platform. The average sequencing depth for all samples in the captured exon regions reached 120X. As for the 96 EBV+ nasopharyngeal carcinoma patients, raw sequence files were obtained from the open database dbGAP‐NHGRI (Study ID: 20 055, Nasopharynx Cancer Whole‐Exome Sequencing). These 96 patients were subjected to exon capture using the SureSelect V2 whole exon kit from Agilent, followed by exome sequencing on the Illumina HiSeq platform, with an average depth of coverage of 80X.

### Single Nucleotide Variation (SNV) and Insertion/Deletion (INDEL) Detection

For the raw sequences, we performed quality control using the fastp software. We enabled the following functions of fastp with default parameters for data preprocessing: 1) Standard filtering function; 2) Automatic identification and trimming of adapter sequences using the built‐in algorithms; 3) Sliding window‐based trimming of low‐quality sequences based on the average quality score; 4) Automatic identification and correction of mismatched bases in overlapping regions of paired‐end reads using base quality recalibration. After preprocessing, we performed quality control analysis using the fastqc software, and all sequencing files passed the quality control tests.

For the clean sequencing files, we used BWA (0.7.17‐r1188) for alignment against the reference genome hg19, employing the BWA‐MEM algorithm. After alignment, we employed the commercial software Sentieon, which utilizes the same mathematical models as the widely accepted GATK software for PCR duplicate removal, base quality control, and identification of SNVs and INDELs. The version of Sentieon solver used was 201 911, and all parameters were set to default. we used Annovar for annotation of the genomic variant call format (VCF) files. The annotation included gene‐based annotation (refGene), region‐based annotation (cytoband), and database filtering‐based annotation (exac03, cosmic70), while all other parameters were set to default.

### Copy Number Variation (CNV) Analysis

For copy number variation analysis, CNVkit to process the tumor BAM files and generate results was utilized. The results were further transformed and reanalyzed using GISTIC 2.0. The analysis results were then exported using maftools (RRID:SCR_02 4519).

### RNA‐seq Analyses

RNA was extracted from nasopharyngeal organoids with integrity number (RIN) ≥ 7.5. RNA‐seq libraries were prepared using NEBNext Ultra RNA Library Prep Kit for Illumina and were sequenced by the Illumina NovaSeq 6000 sequencing machine with 150‐bp paired‐end reads. The RNA‐seq reads were aligned to the reference genome (GRCm38) by STAR_2.6.0a. Transcript abundance was normalized and measured by Transcript per million (TPM). Differential gene expression was analyzed by DESeq2 (RRID:SCR_01 5687) and pathway network analyses were performed by clusterProfiler (RRID:SCR_01 6884). Genes with an absolute fold change greater than 1 and *p*.adj ≤ 0.05 were counted as differentially expressed genes. The heatmaps of differentially expressed genes were done by pheatmap_1.0.12 and normalized by Z scores. The TPM data were used for GSEA. GSEA was employed to determine statistically significant similarities and differences between two given clusters by identifying prior‐defined gene sets. ggpubr_0.4.0 was used to portray the box plot. *P* values were calculated by t‐test. Venn diagrams were generated with the R package of VennDiagram_1.6.20. The gene knockout efficiency was plotted by Integrative Genomics Viewer (IGV, RRID:SCR_01 1793).

### Statistical Analyses

The statistical test methods, sample sizes, and *p*‐values involved in this article were indicated in the corresponding legends. Statistical analyses were performed using GraphPad Prism 7.0 (RRID: SCR_0 02798). To determine statistical probabilities, the student's t‐test was performed where appropriate, and *p* < 0.05 was considered statistically significant. Data were presented as the Mean ± SEM.

## Conflict of Interest

The authors declare no conflict of interest.

## Author Contributions

X.W., Y.L., Y.P., J.W., and S.M.Y. contributed equally to this work. X.W., Y.L., Y.P., J.W., S.Y., designed and performed experiments, analyzed data and wrote the manuscript. W.W., K.R., H.L., Y.L., Q.Y., L.Z., D.L., P.L., S.L., L.L., L.G., J.D., M.Z., S.D., Y.Y, H.L., participated in the experiments and analyzed date. X.W., Y.L., Y.P., L.Z., L.Z., X.C., performed bioinformatics analyses. Q.Y., performed histopathological analysis. N.C., J.B., L.F., Y.W., supervised the study, provided resources, and analyzed data. Y.L., M.Z, C.C., and Q.Z., conceived the project, designed experiments, analyzed data and wrote the manuscript.

## Supporting information

Supporting Information

Supporting Information

Supporting Information

Supporting Information

Supporting Information

Supporting Information

Supporting Information

## Data Availability

The data that support the findings of this study are openly available in National Genomics Data Center, China National Center for Bioinformation/Beijing Institute of Genomics, Chinese Academy of Sciences at http://bigd.big.ac.cn/gsa‐human, reference number 5496.
