# Supplementary material for: Primary and Orthotopic Murine Models of Nasopharyngeal Carcinoma Reveal Molecular Mechanisms Underlying its Malignant Progression
Source: Adv Sci (Weinh). 2024 Jul 25;11(36):2403161. doi: 10.1002/advs.202403161 (PMC11423139; doi:10.1002/advs.202403161)
Supplement: Supplementary file 1 — Supporting Information [file ADVS-11-2403161-s002.docx]

**Supplementary Figures**


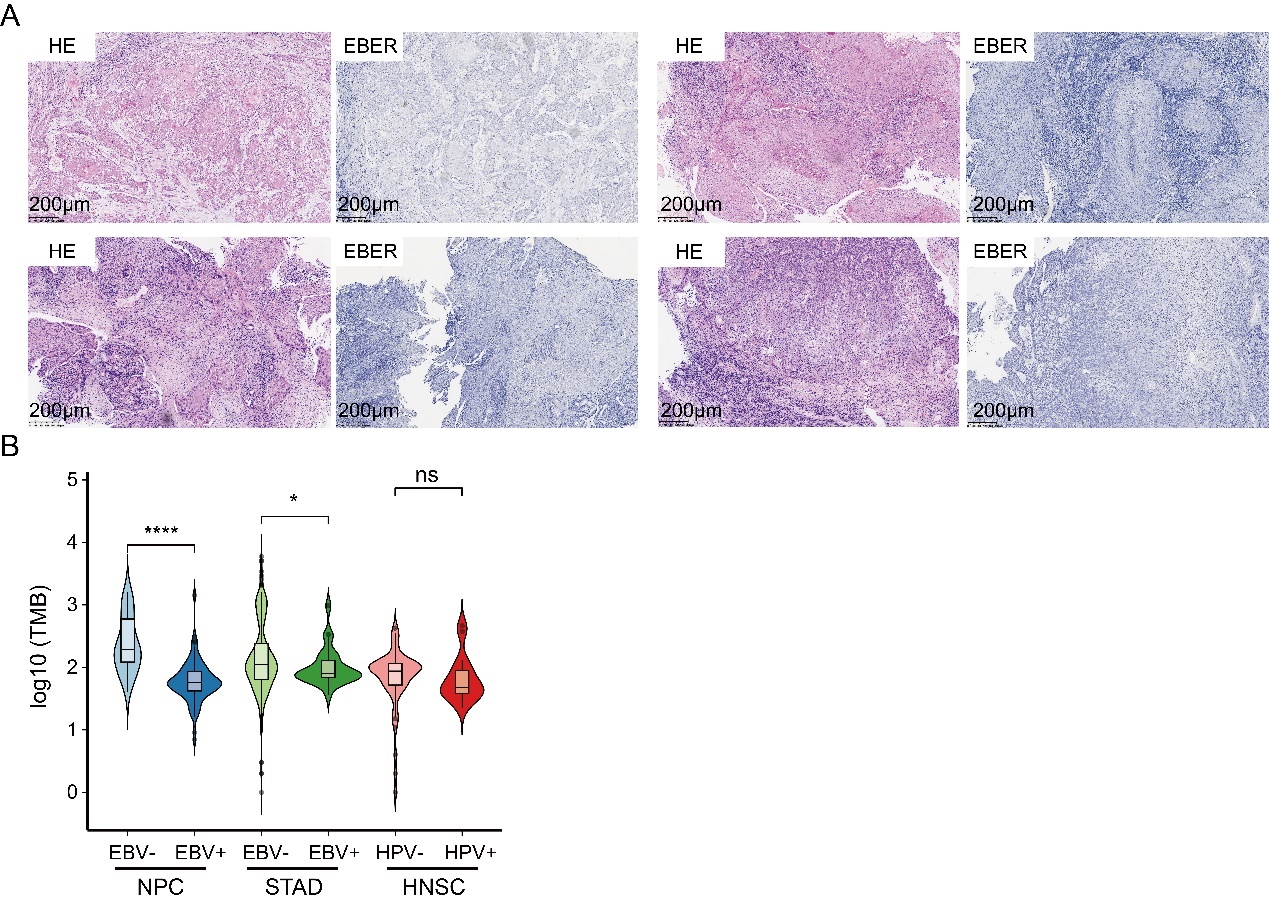
**Figure S1**

(A) Representative H&E staining and ISH staining for EBER in EBV- NPCs. Scale bar, 200 μm.

(B) Violin plot showing the tumor mutation burden (TMP) for EBV+ and EBV- NPC, STAD and HNSC patients. EBV- NPC(n=32), EBV+NPC(n=96), EBV-STAD(n=411), EBV+STAD(n=26), HPV-HNSC(n=57), HPV+HNSC(n=37). (Wilcox. Test, ns, *p* > 0.05; *, *p* <= 0.05; ****, *p* <= 0.0001).


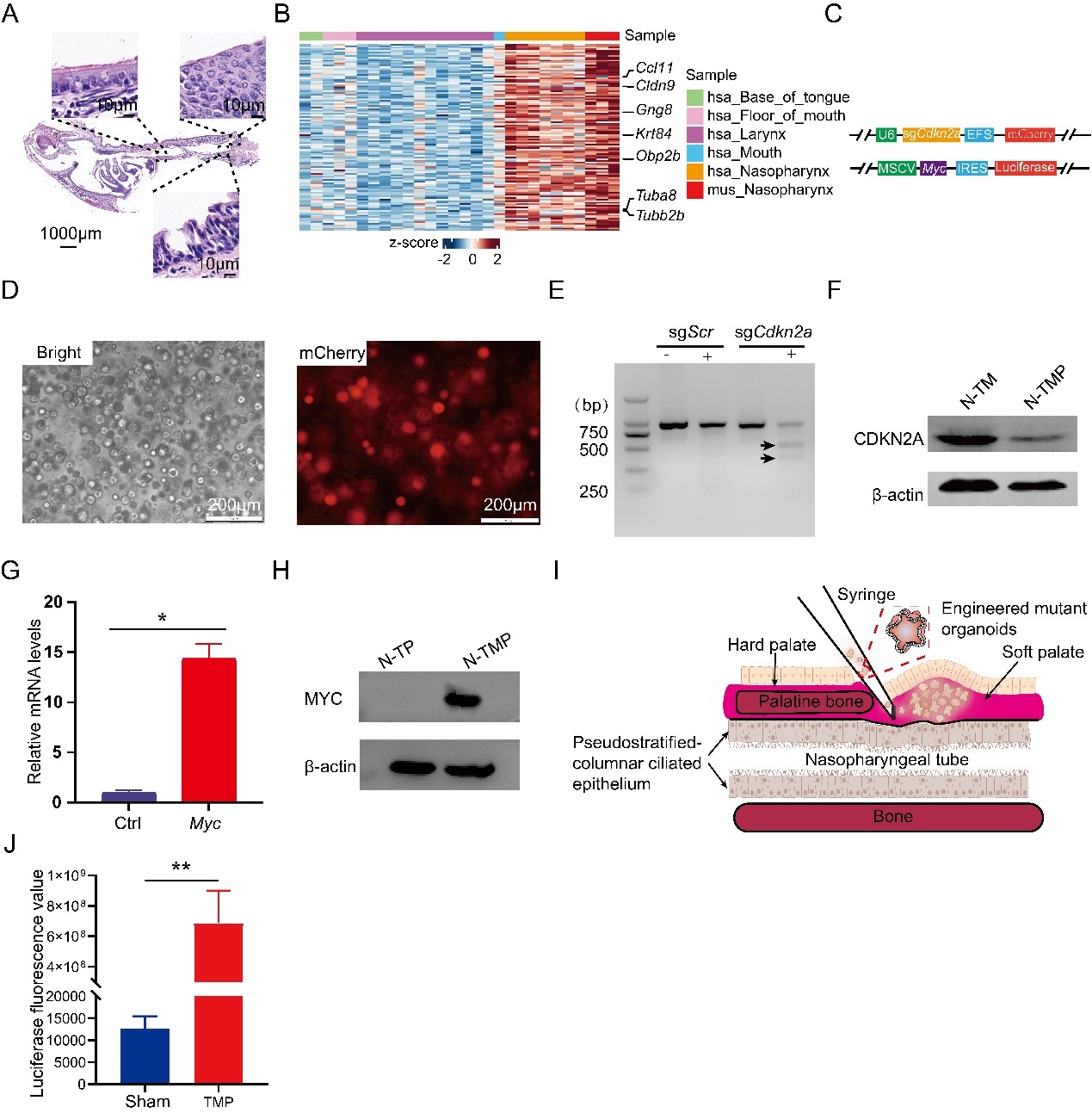
**Figure S2.**

(A) Representative H&E images of mouse nasal airway epithelium. Scale bar, 10μm.

(B) Heatmap showing the up-regulated genes in mouse nasopharyngeal organoids and human nasopharyngeal epithelium compared with other human head and neck epithelium.

(C) Schematic of the constructs for targeting *Cdkn2a* and overexpression of *Myc*.

(D) Highly efficient virus infection in Cas9-expressing *Trp53*-/- Organoids. Scale bar, 200μm.

(E) Analysis of *Cdkn2a* mutation in premalignant organoids using T7 enzyme digestion method.

(F) Western blots showing the *Cdkn2a* levels in TM and TMP premaligant nasopharyngeal organoids.

(G) QRT-PCR analysis for expression of *Myc* in organoids transduced a control construct and organoids transduced the construct for expressing *Myc*. *, *p*< 0.05.

(H) Western blots showing the MYC levels in organoids transduced a control construct and organoids transduced the construct for expressing *Myc*.

(I) Schematic diagram showing the method of detailed orthotopic transplantation.

(J) Comparison of luciferase values of mice transplanted with Matrigel and TMP premalignant organoids. n=8 animals per group. **, *p*<0.01.


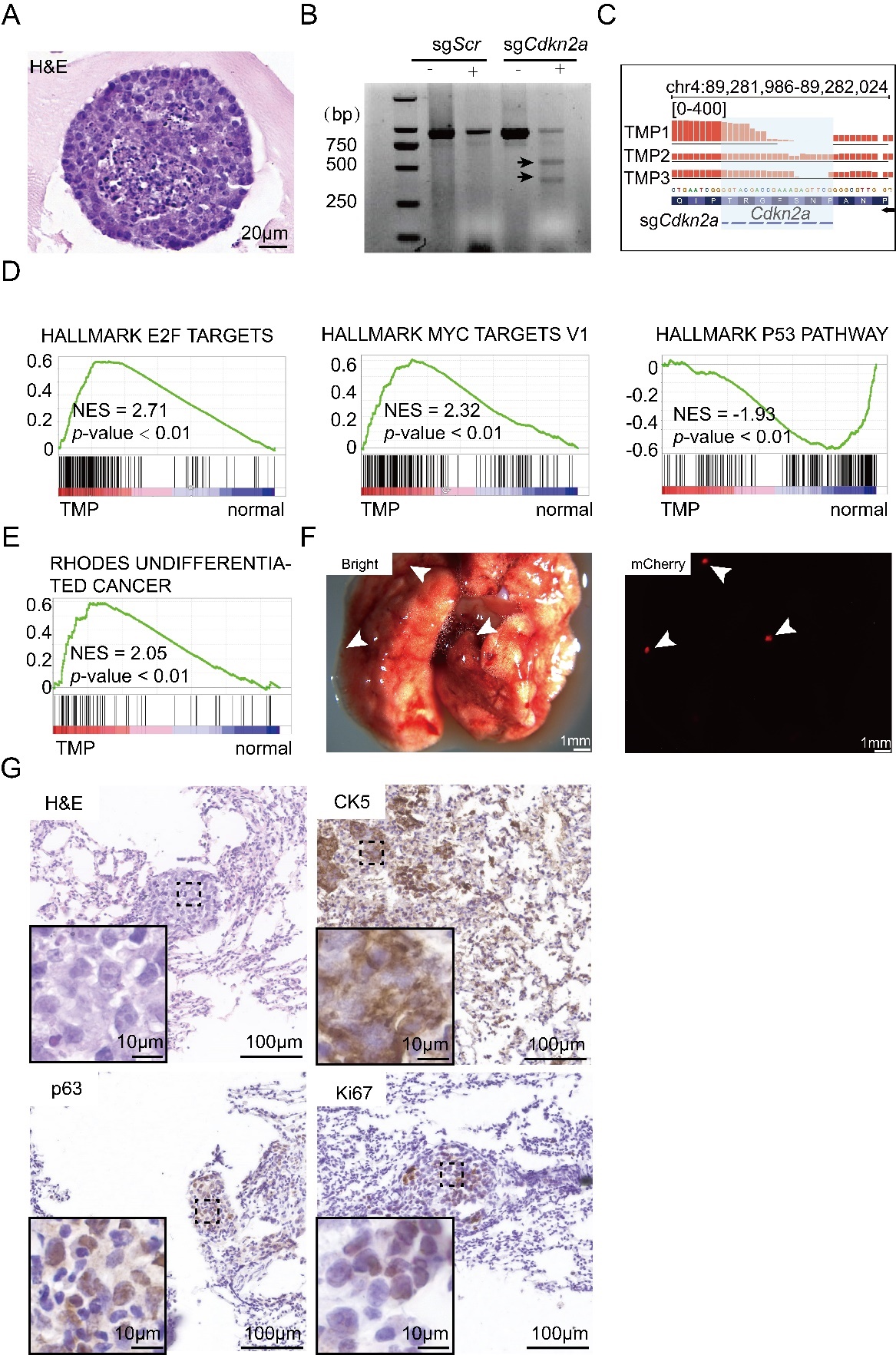
**Figure S3.**

(A) Representative H&E image of tumor organoids established from TMP primary and orthotopic tumor tissues. Scale bar, 20μm.

(B) Analysis of *Cdkn2a* mutation in TMP primary and orthotopic tumors using T7 enzyme digestion method.

(C) The IGV plot showing *Cdkn2a* mutations in the TMP tumors at the sg*Cdkn2a* targeted regions.

(D) GSEA showing enrichment of the gene sets of “HALLMARK E2F TARGETS”, “HALLMARK MYC TARGETS V1”, and “HALLMARK P53 PATHWAY” in the TMP tumor organoids, compared to normal nasopharygneal organoids.

(E) GSEA showing positive enrichment of “RHODES UNDIFFERENTIATED CANCER” in the TMP tumor organoids, compared to normal nasophangeal organoids.

(F) Representative images of macroscopic lung metastasis for TMP genotype in nude mice. Bright image of lung lobes (left). mCherry-fluorescent metastasized tumors in lung lobes (right). Scale bar, 1mm.

(G) ) Representative H&E staining of metastasized lung tumors in nude mice. Scale bar, 100μm. Representative IHC staining of metastasized lung tumors in nude mice. Scale bar, 100μm.


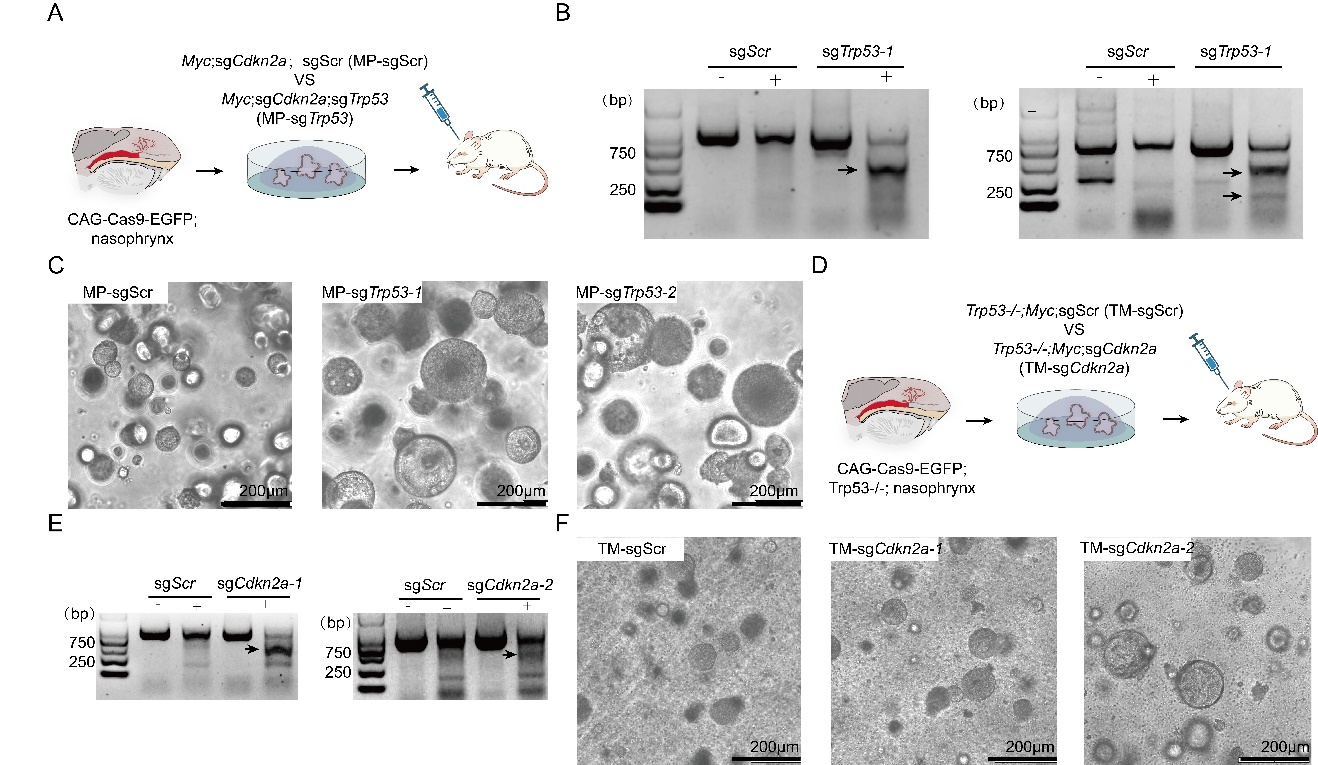
**Figure S4.**

(A) Schematic diagram of the strategy for exploring functions of *Trp53* loss with premalignant gene-edited organoids in nude mice.

(B) Analysis of *Trp53* mutation in premalignant organoids using T7 enzyme digestion method.

(C) Representative images of MP-sgScr, MP-sg*Trp53-1* and MP-sg*Trp53-2* premalignant organoids. Scale bar, 200μm.

(D) Schematic diagram of the strategy for exploring functions of *Cdkn2a* loss with premalignant gene-edited organoids in nude mice.

(E) Analysis of *Cdkn2a* mutation in premalignant organoids using T7 enzyme digestion method.

(F) Representative images of TM-sgScr, TM-sg*Cdkn2a-1* and TMP-sg*Cdkn2a-2* in premalignant organoids. Scale bar, 200μm.


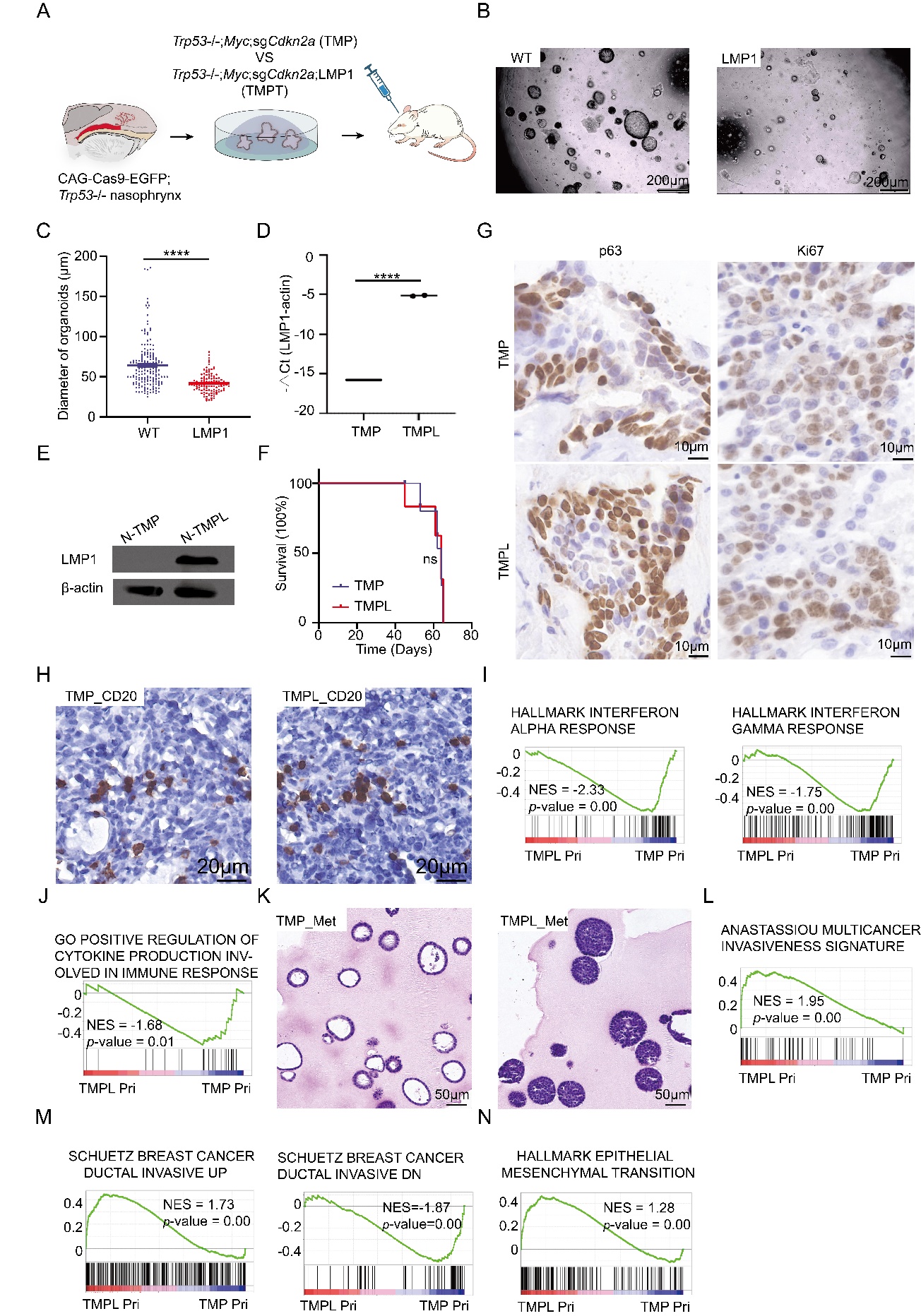
**Figure S5.**

(A) Schematic diagram of the strategy for exploring functions of LMP1 with premalignant gene-edited organoids in nude mice.

(B) Representative images of normal nasopharyngeal organoids and organoids transduced the construct for expressing LMP1. Scale bar, 200μm.

(C) Comparison of sizes between normal nasopharyngeal organoids and organoids transduced the construct for expressing LMP1. ****, *p* < 0.0001. Data are means ± SEM. WT (n=174); LMP1 (n=113).

(D) QRT-PCR analysis for expression of LMP1 in TMP organoids and TMPL premalignant organoids. n=2 samples per group. ****, *p* < 0.0001.

(E) Immunoblots of LMP1 in the TMP and TMPL premalignant organoids.

(F) Survival curve of nude mice orthotopically transplanted with TMP and TMPL premalignant organoids. n=4 animals per group.

(G) Representative IHC staining of TMP and TMPL primary tumors for p63 and Ki67. Scale bar, 10μm.

(H) Representative IHC staining of in TMP and TMPL primary tumors for CD20 in nude mice. Scale bar, 20μm.

(I) GSEA showing enrichment of gene sets of “HALLMARK INTERFERON ALPHA RESPONSE” and “HALLMARK INTERFERON GAMMA RESPONSE” in TMPL primary tumors, compared with TMP primary tumors.

(J) GSEA showing enrichment of gene sets of “POSITIVE REGULATION OF CYTOKINE PRODCTION INVOLVED IN IMMUNE RESPONSE” in TMPL primary tumors, compared with TMP primary tumors.

(K) H&E staining of tumor organoids derived from TMP and TMPL metastasized tumors. Scale bar, 50μm.

(L) GSEA showing enrichment of gene sets of “ANASTASSIOU MULTICANCER INVASIVENESS SIGNATURE” in the TMPL tumor organoids, compared to TMP primary organoids.

(M) GSEA showing enrichment of gene sets of “SCHUETZ BREAST CANCER DUCTAL INVASIVE UP” and “SCHUETZ BREAST CANCER DUCTAL INVASIVE UP” in TMPL primary tumors, compared with TMP primary tumor.

(N) GSEA showing positive enrichment of “HALLMARK EPITHELIAL MESENCHYMAL TRANSITION” in the TMPL tumor organoids, compared to TMP paimary organoids.


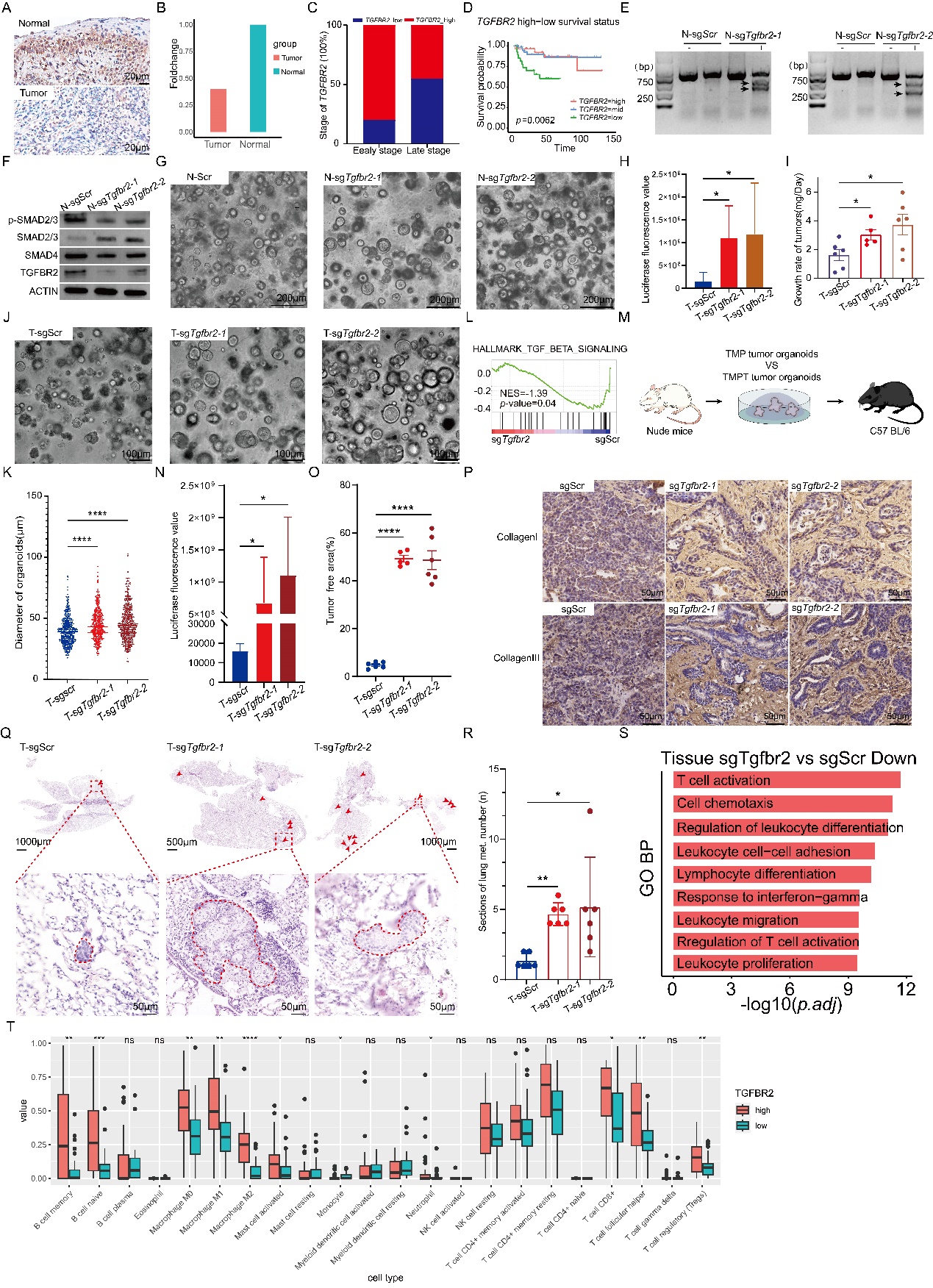
**Figure S6.**

(A) Comparison of *TGFBR2* expression in human NPCs, compared to corresponding normal tissues. Scale bar, 20μm.

(B) Representative cases showing down expression of *TFGBR2* in human NPCs, compared to normal human nasopharyngeal epithelium. Normal Samples(n=18), Tumor Samples(n=18).

(C) Comparison of proportion of patients with high *TGFBR2* or low *TGFBR2* expression in early stage and late stage, respectively.

(D) Survival curve of NPC patients with high, middle, and low expression of *TGFBR2*. Group high(n=28), Group mid(n=56), Group low(n=28).

(E) Analysis of *Tgfbr2* mutation in gene-edited premalignant organoids using T7 enzyme digestion method.

(F) Western blots showing the TGFβ pathway-related components in premalignant TMP and TMPT organoids.

(G) Representative images of premalignant TMP and TMPT organoids. Scale bar, 200μm.

(H) Comparison of luciferase fluorescence values of mice transplanted with TMP and TMPT premalignant organoids. T-sgScr (n=6); T-sg*Tgfbr2-1* (n=5); T-sg*Tgfbr2-2* (n=6). *, *p*<0.05.

(I) Comparison of growth rate of TMP and TMPT tumors in nude mice. T-sgScr (n=6); T-sg*Tgfbr2-1* (n=5); T-sg*Tgfbr2-2* (n=6). *, *p*<0.05

(J) Representative images of tumor organoids established from TMP and TMPT primary and orthotopic tumors. Scale bar, 100μm.

(K) Comparison of sizes between TMP and TMPT tumor organoids. ****, *p* < 0.0001. Data are means ± SEM. sgScr (n=535), sg*Tgfbr2-1* (n=522), and sg*Tgfbr2-2* (n=518).

(L) GSEA showing enrichment of gene sets of “HALLMARK TGF BATE SIGNALING” in TMPT primary tumors, compared with TMP primary tumors.

(M) Schematic diagram of the strategy for exploring functions of *TGFBR2* loss with tumor organoids in C57BL/6 mice.

(N) Comparison of luciferase fluorescence values of C57BL/6 mice transplanted with TMP and TMPT tumor organoids after 55 days for transplantation. n=6 animals per group. *, *p*<0.05.

(O) Comparison of stromal areas of TMP and TMPT tumors in nude mice. T-sgScr (n=6); T-sg*Tgfbr2-1* (n=5); T-sg*Tgfbr2-2* (n=6). ****, *p*<0.0001.

(P) Representative IHC staining of TMP and TMPT primary tumors for collagen I and collagen III. Scale bar, 50μm.

(Q) The representative H&E staining of the lung section of TMP and TMPT NPC mice. Scale bar, 50μm.

(R) Comparison of the number of metastatic lung lesions in lung sections of TMP and

TMPT NPC mice. n=6 sections per group. *, *p*<0.05, **, *p* < 0.01.

(S) Bar plots showing the pathways related to immune response in TMPT tumors, compared to TMP tumors in nude mice.

(T) The infiltration state of immune related cells in *TGFBR2*_high and *TGFBR2*_low NPC patients. Group high(n=28), Group low(n=28). “*”, *p*<0.05, “**”, *p*<0.01, ****, *p*<0.0001.
